# Off-Hour Effect on 3-Month Functional Outcome after Acute Ischemic Stroke: A Prospective Multicenter Registry

**DOI:** 10.1371/journal.pone.0105799

**Published:** 2014-08-28

**Authors:** Chulho Kim, Min Uk Jang, Mi Sun Oh, Jong-Ho Park, San Jung, Ju-Hun Lee, Kyung-Ho Yu, Moon-Ku Han, Beom Joon Kim, Tai Hwan Park, Sang-Soon Park, Kyung Bok Lee, Jae Kwan Cha, Dae-Hyun Kim, Jun Lee, Sung-Hun Kim, Soo Joo Lee, Youngchai Ko, Jong-Moo Park, Kyusik Kang, Young-Jin Cho, Keun-Sik Hong, Ki-Hyun Cho, Joon-Tae Kim, Dong-Eog Kim, Jay Chol Choi, Myung Suk Jang, Hee-Joon Bae, Byung-Chul Lee, on the behalf of CRCS-5 investigators

**Affiliations:** 1 Department of Neurology, Hallym University Sacred Heart Hospital, Chuncheon-si, Republic of Korea; 2 Department of Neurology, Hallym University Sacred Heart Hospital, Anyang-si, Republic of Korea; 3 Department of Neurology, Myongji Hospital, Goyang-si, Republic of Korea; 4 Department of Neurology, Kangnam Sacred Heart Hospital, Seoul, Republic of Korea; 5 Department of Neurology, Kangdong Sacred Heart Hospital, Seoul, Republic of Korea; 6 Department of Neurology and Cerebrovascular Center, Seoul National University Bundang Hospital, Seongnam-si, Gyeonggi-do, Republic of Korea; 7 Department of Neurology, Seoul Medical Center, Seoul, Republic of Korea; 8 Department of Neurology, Soonchunhyang University Hospital, Seoul, Republic of Korea; 9 Department of Neurology, Dong-A University Hospital, Busan, Republic of Korea; 10 Department of Neurology, Yeungnam University Medical Center, Daegu, Republic of Korea; 11 Department of Neurology, College of Medicine, Kangwon National University, Chuncheon-si, Republic of Korea; 12 Department of Neurology, Eulji University Hospital, Daejeon, Republic of Korea; 13 Department of Neurology, Eulji General Hospital, Seoul, Republic of Korea; 14 Department of Neurology, Inje University Ilsan Paik Hospital, Goyang-si, Gyeonggi-do, Republic of Korea; 15 Department of Neurology, Chonnam National University Hospital, Gwangju, Republic of Korea; 16 Department of Neurology, Dongguk University Ilsan Hospital, Goyang-si, Gyeonggi-do, Republic of Korea; 17 Department of Neurology, Jeju National University Hospital, Jeju-do, Republic of Korea; Cardiff University, United Kingdom

## Abstract

**Background and Purpose:**

The time of hospital arrival may have an effect on prognosis of various vascular diseases. We examined whether off-hour admission would affect the 3-month functional outcome in acute ischemic stroke patients admitted to tertiary hospitals.

**Methods:**

We analyzed the ‘off-hour effect’ in consecutive patients with acute ischemic stroke using multi-center prospective stroke registry. Work-hour admission was defined as when the patient arrived at the emergency department between 8 AM and 6 PM from Monday to Friday and between 8 AM and 1 PM on Saturday. Off-hour admission was defined as the rest of the work-hours and statutory holidays. Multivariable logistic regression was used to analyze the association between off-hour admission and 3-month unfavorable functional outcome defined as modified Rankin Scale (mRS) 3–6. Multivariable model included age, sex, risk factors, prehospital delay time, intravenous thrombolysis, stroke subtypes and severity as covariates.

**Results:**

A total of 7075 patients with acute ischemic stroke were included in this analysis: mean age, 67.5 (±13.0) years; male, 58.6%. In multivariable analysis, off-hour admission was not associated with unfavorable functional outcome (OR, 0.89; 95% CI, 0.72–1.09) and mortality (OR, 1.09; 95% CI, 0.77–1.54) at 3 months. Moreover, off-hour admission did not affect a statistically significant shift of 3-month mRS distributions (OR, 0.90; 95% CI, 0.78–1.05).

**Conclusions:**

‘Off-hour’ admission is not associated with an unfavorable 3-month functional outcome in acute ischemic stroke patients admitted to tertiary hospitals in Korea. This finding indicates that the off-hour effects could be overcome with well-organized stroke management strategies.

## Introduction

Stroke is one of the leading cause of death in Korea, with an estimated annual incidence of 105000 [1]. The prognosis of acute ischemic stroke is affected by age [2], stroke severity [3], infarct location [4], comorbid conditions such as hyperglycemia [5], low hemoglobin levels [6], renal dysfunction [7], and timing of hospital admission [8]–[22]. Previous studies suggested that stroke patients admitted during the weekend were more likely to have a worse functional outcome and higher mortality compared to those admitted on weekdays [21], [22]. This weekend effect may be explained by reduced hospital staffing, delays in diagnostic procedures, and a low rate of thrombolysis during the weekend period [21]–[24]. However, stroke admissions on off-hour of weekdays as well as weekends are at risk of reduced access to timely and appropriate management [13]. Therefore, rather than only the weekend effect, the off-hour effect on outcome should be explored to assess whether consistent cares area provided to patients with acute ischemic stroke.

Numerous studies showed that organized stroke care consistently improves mortality rates and functional outcomes in patients with ischemic stroke [25]–[27]. Organized stroke care needs multidisciplinary teams including physicians, interventionists, specialized nurses, rehabilitation staffs, and coordinating personnel and that are readily available at all times. Therefore, the clinical outcome may be consistent in patients receiving well-organized stroke care irrespective of the timing of hospital admission.

The purpose of this study was to examine whether off-hour admission has an impact on 3-month functional outcome in ischemic stroke patients in tertiary hospitals.

## Methods

We used the database of Clinical Research Center for Stroke (CRCS) registry-5, which is a hospital-based multi-center prospective registry for acute stroke patients. The CRCS-5 registry was established in April 2008 and contains demographic and clinical data of consecutive patients with acute ischemic stroke admitted within 7 days of onset. Twelve tertiary teaching hospitals in Korea have participated in the CRCS-5 registry, all of which have a comprehensive stroke center [28].

**Figure 1:**
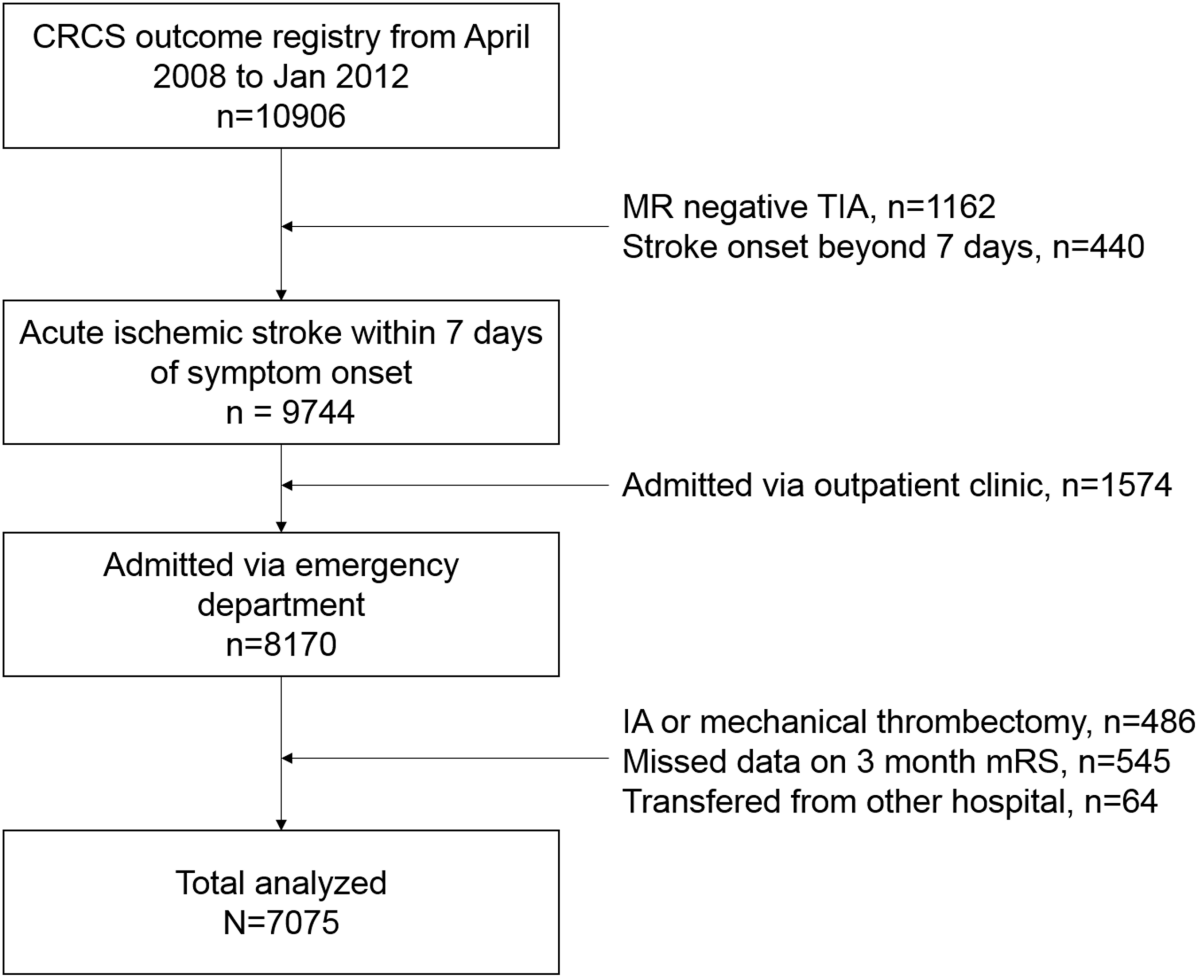
Flow Diagram for Study Subjects.

Eligible patients for this study had an ischemic lesion on MRI corresponding to acute stroke symptoms within 7 days of symptom onset. In Korea, brain MR imaging is routinely performed in patients who are tentatively diagnosed with acute ischemic stroke in academic stroke center. Figure 1 shows flow diagram for study subjects. From April 2008 to January 2012, a total of 10906 patients with acute ischemic stroke were registered into the CRCS database. We excluded patients who were admitted via outpatient clinic or transferred from the other hospital. Moreover, patients who received intra-arterial thrombolysis or mechanical thrombectomy (n = 486), because substantial disparity in the performance rate of interventional recanalization therapy (intra-arterial thrombolysis or mechanical thrombectomy) among participating hospitals could influence patients’ clinical outcome and hospital selection. Additionally, our participating hospitals are tertiary referral center. Among referred patients, there were some portion of patients transferred from other hospital for the interventional therapy, which would be influence the timing of hospital arrival.

Work-hour admission was defined as an arrival at the emergency department between 8 AM and 6 PM from Monday to Friday and between 8AM and 1PM on Saturday. Off-hour admission was defined as the rest of the work-hours and statutory holidays.

Subjects were considered hypertensive if they were taking antihypertensive medications, if their average sitting systolic blood pressure was 140 mmHg or more, of if their diastolic blood pressure was 90 mmHg or more. Subjects were diagnosed with diabetes if they were taking medical treatments for diabetes, if they had a fasting serum glucose level of 126 mg/dL or more, or if they had a non-fasting random serum glucose level of 200 mg/dL or more with corresponding symptoms of diabetes. Subjects were considered to have hyperlipidemia if they had a fasting total cholesterol level of 240 mg/dL or more, or if they were being treated with a lipid-lowering agent. A current smoker was defined as a person smoking one or more cigarettes per day within the last 6 months.

Descriptive comparisons between the off-hour and work-hour groups were examined using the χ^2^ test or Mann-Whitney *U* test for categorical variables, and *Student t* test for continuous variables, as appropriate. Cross-sectional comparison between the groups for unfavorable functional outcome (modified Rankin Scale score; mRS>2) and death at 3 months were conducted using a multiple logistic regression model. We used a multivariable ordinal logistic regression analysis for the shift to the unfavorable functional outcome (ie, shift of mRS from 0 to 1) at 3 months, taking the 7-categorical mRS scores as a dependent variable. Covariates having *P*<0.10 in each univariable analysis were selected for input into the multivariable models. Forced entry with the off- and work-hour groups was used in multivariable analysis. Two-sided *P*<0.05 was considered significant. Repeated statistics for ischemic stroke patients with IV thrombolysis was performed as a sensitivity analysis.

The design of the study was approved by Hallym University Sacred Heart Hospital Institutional Review Board/Ethic Committee. Written informed consent for the registry enrollment and 3-month outcome capture was given by participants (or next of kin). Moreover, patients’ clinical informations were stored anonymously in the registry server and we extracted deidentified data prior to analysis.

## Results

The mean age (± SD) and proportion of men in the study population were 67.5±13.0 years and 58.6% (4146 of 7075), respectively. The number of patients admitted to an emergency department during off-hours was 3473 (49.1%). Baseline characteristics of the included subjects are described in Table 1. Compared to the work-hour group, the off-hour group were younger and had higher National Institute of Health Stroke Scale (NIHSS) scores, less frequent history of diabetes, shorter prehospital delay times, and had received more thrombolytic treatments. Among patients treated with IV thrombolysis, the door-to-needle time was comparable between the off-hour and work-hour groups, and it was less than 60 minutes in the two groups. Table S1 showed baseline characteristics of patients with (n = 7075) and without (n = 532) 3-month mRS. Although patients with missing mRS had a higher proportion of hyperlipidemia (44.7% vs. 33.0%, *P* = 0.001) and a higher stroke severity (median 6 vs. 3, *P*<0.001) compared with patients with mRS, proportions of off-hour admission and prehospital delay times were not statistically different between the two groups.

**Table 1:** Baseline Characteristics of Study Subjects.

	Total(n = 7075)	Work-hours(n = 3602)	Off-hours(n = 3473)	*P* value
Age, mean (±SD)	67.5 (±13.0)	67.8 (±12.7)	67.2 (±13.3)	0.040
Male, n (%)	4146 (58.6)	2133 (59.2)	2013 (58.0)	0.284
Risk Factor, n (%)				
Previous stroke	1518 (21.5)	790 (21.9)	728 (21.0)	0.320
Hypertension	4926 (69.6)	2528 (70.2)	2398 (69.0)	0.299
Diabetes	2379 (33.6)	1254 (34.8)	1125 (32.4)	0.031
Hyperlipidemia	2334 (33.0)	1168 (32.4)	1166 (33.6)	0.305
Current smoking	1875 (26.5)	949 (26.3)	926 (26.7)	0.763
TIA presentation andStroke subtype, n (%)				0.028‡
TIA presentation	35 (0.5)	21 (0.6)	14 (0.4)	
LAA	2608 (36.9)	1374 (38.1)	1234 (35.5)	
SVO	1365 (19.3)	714 (19.8)	651 (18.7)	
CE	1416 (20.0)	687 (19.1)	729 (21.0)	
SOE	176 (2.5)	80 (2.2)	96 (2.8)	
SUE	1475 (20.8)	726 (20.2)	749 (21.6)	
NIHSS score at admission				
Mean (±SD)	5.4 (±5.7)	5.2 (±5.5)	5.6 (±6.0)	0.001
Median (IQR)	3 (5)	3 (6)	4 (6)	0.007
Prehospital delay, hour* †	8.6 (26.4)	14.7(40.0)	6.3 (18.0)	<0.001
Onset to Needle time, min†	115.0 (62.5)	119.0 (70.0)	114.0 (61.0)	0.737
Door to Needle time, min†	45.0 (22.3)	44.0 (22.5)	45.0 (23.5)	0.082
IV rtPA	606 (8.6)	265 (7.4)	341 (9.8)	<0.001

Values provided are expressed as number (%) or mean ± SD (standard deviation), as appropriate, or otherwise stated.

*The time from onset of stroke symptom to hospital visit.

† median (IQR, interquartile range).

‡ *χ* ^2^ test.

TIA: transient ischemic attack; LAA, large artery atherosclerosis; SVO, small vessel occlusion; CE, cardioembolism; SOE, stroke of other determined etiology; SUE, stroke of undetermined etiology; IV: intravenous, rtPA: recombinant tissue plasminogen activator; NIHSS, National Health Institute Stroke Scale.

Among the 7075 participants, a total of 4449 patients (62.9%) had a favorable outcome (mRS, 0–2) at 3 months after stroke onset (Table 2). The proportions of patients with 3-month mRS score of 3–6 were 37.2% in the off-hour group and 37.1% in the work-hour group, and the difference was not statistically significant in univariable analysis.

**Table 2:** Univariable and Multivariable Analysis for Unfavorable Functional Outcome at 3 Months (mRS, 3–6).

	Unfavorable outcome(n = 2626)	Favorable outcome(n = 4449)	Univariable analysis		Multivariable analysis	
			OR (95% CI)	*P*	*aOR (95% CI)	*P*
Age, year	72.8 (±11.6)	64.4 (±12.8)	1.06 (1.06–1.07)	<0.001	1.05 (1.04–1.06)	<0.001
Male	1281 (48.8)	2865 (64.4)	0.53 (0.48–0.58)	<0.001	0.68 (0.55–0.85)	0.001
Risk factor (%)						
Previous stroke	824 (31.4)	694 (15.6)	2.47 (2.20–2.78)	<0.001	2.14 (1.68–2.73)	<0.001
Hypertension	1941 (73.9)	2985 (67.1)	1.39 (1.25–1.55)	<0.001	0.93 (0.74–1.18)	0.539
Diabetes	989 (37.7)	1390 (31.2)	1.33 (1.20–1.47)	<0.001	1.12 (0.90–1.40)	0.311
Hyperlipidemia	850 (32.4)	1484 (33.4)	0.96 (0.86–1.06)	0.394		
Current Smoking	511 (19.5)	1364 (30.7)	0.55 (0.49–0.61)	<0.001	1.10 (0.84–1.44)	0.496
TIA presentation andStroke subtype, n (%)						
TIA presentation	3 (0.1)	32 (0.7)	0.35 (0.11–1.15)	0.084	0.01 (1.00–1.00)	1.000
LAA	1023 (39.0)	1585 (35.6)	2.41 (2.07–2.81)	<0.001	1.74 (1.30–2.32)	<0.001
SVO	288 (11.0)	1077 (24.2)	1.0 (referent)	-	1.0 (referent)	-
CE	685 (26.1)	731 (16.4)	3.50 (2.97–4.14)	<0.001	1.02 (0.72–1.45)	0.898
SOE	59 (2.2)	117 (2.6)	1.89 (1.34–2.65)	<0.001	4.37 (2.33–8.21)	<0.001
SUE	568 (21.6)	907 (20.4)	2.34 (1.98–2.77)	<0.001	1.18 (0.84–1.64)	0.345
NIHSS score at admission						
Mean (±SD)	9.3 (±6.8)	3.1 (±3.3)	1.31 (1.29–1.33)	<0001	1.29 (1.25–1.32)	<0.001
Median (IQR)	7 (10)	2 (3)		<0.001		
Prehospital delay (hour)	7.7 (25.2)	9.1 (±27.6)	1.00 (1.00–1.00)	0.005	1.00 (1.00–1.00)	0.935
Onset to Needle time (min)	115 (67)	115 (62)		0.906		
Door to Needle time (min)	45 (24)	45 (22)		0.366		
IV rtPA	282 (29.2)	324 (23.4)	1.35 (1.12–1.63)	0.002	0.46 (0.34–0.61)	<0.001
Off-hour (vs. Work-hour)	1291 (49.2)	2182 (49.0)	1.01 (0.91–1.11)	0.924	0.89 (0.72–1.09)	0.262

Abbreviations are presented in the previous table.

All the 7075 patients were included in the multivariable analysis.

*aOR: adjusted odds ratio.

Among covariates, univariable analyses showed that age, sex, prior stroke history, diabetes, smoking, stroke subtype, initial NIHSS score, intravenous thrombolysis, and prehospital delay times were associated with 3-month mRS outcome of 3–6. Even after adjusting for these covariates, off-hour admission was not associated with unfavorable functional outcome (OR, 0.89; 95% CI, 0.72–1.09, *P* = 0.262). Advanced age (OR, 1.05 per 1 year increase; 95% CI, 1.04–1.06), male gender (OR, 0.68; 95% CI, 0.55–0.85), prior stroke history (OR, 2.14; 95% CI, 1.68–2.73), initial stroke severity (OR, 1.29 per 1 point NIHSS score increase; 95% CI, 1.25–1.32), and IV thrombolysis (OR, 0.46; 95% CI, 0.34–0.61) were independent predictors for unfavorable functional outcome at 3 months.

A total of 427 patients out of 7075 subjects died within 3 months of symptom onset (Table S2). In univariable analysis, off-hour admission was associated with 3-month mortality. However, its difference was not statistically significant after adjusting covariates (OR versus work-hour, 1.09; 95% CI, 0.77–1.54). In shift analysis, off-hour admission did not affect a statistically significant shift of 3-month mRS distributions (Table S3). Table 3 summarized the association between off-hour admission and unfavorable functional outcome according to each multivariable models. In addition, an analysis restricted to patients with IV thrombolysis also showed that off-hour admission was not associated with 3-month unfavorable functional outcome, whereas higher age and stroke severity were independent predictors of unfavorable functional outcomes (Table S4).

**Table 3:** A Comparison of Association Between Off-hour Admission and Each Outcome at 3 Months from Multivariable Analyses.

Methods of analysis	OR (95% CI)	*P* value
Unfavorable functional outcome(dichotomized, 3–6 vs. 0–2)		
Off-hour	0.89 (0.72–1.09)	0.262
Mortality (dichotomized)		
Off-hour	1.09 (0.77–1.54)	0.608
Change in the distribution ofmRS score (shift analysis)		
Off-hour	0.90 (0.78–1.05)	0.187
Unfavorable functional outcome in patientswith IV thrombolysis(subgroup, dichotomized, 3–6 vs. 0–2)		
Off-hour	0.85 (0.58–1.25)	0.428

Abbreviations are presented in the previous table.

Finally, we analyzed 4940 patients admitted within the first 24 hours from stroke onset (Table S5). In multivariable analysis, off-hour admission was not a predictor for 3-month poor outcome (OR, 0.89; 95% CI, 0.70–1.13, *P* = 0.335). Moreover, result was not changed by the onset to admission time (within 24 hours and within 7 days of onset of symptoms).

## Discussion

In our study, off-hour admission was not associated with unfavorable 3-month functional outcome in acute ischemic stroke patients, although stroke severity was even higher in patients admitted during off-hours than in those admitted during work-hours. Moreover, results of 3-month mortality and analyses for a shift of mRS distributions showed that off-hour admission was not associated with worse prognosis at 3 months.

Besides the main findings, other aspects of the data are worth considering for the off-hour effect on prognosis of acute ischemic stroke. In addition to the differences in age, stroke risk factors, and stroke subtypes between the off-hour and the work-hour admission groups, stroke severity and the rate of IV thrombolysis were higher, while prehospital delay time was shorter in patients admitted during off-hours than in those admitted during work-hours. Moreover, door to needle time was not significantly longer in the off-hour admission group than in the work-hour admission group. The findings indicate that fast distribution of medical resources, even during off-hour period, occurs in well-organized stroke centers. Efficient placement of medical personnel, timely operation of a critical pathway, and appropriate laboratory delivery may be among the reasons why a negative off-hour effect was not found in these recently recruited prospective stroke data. However, this hypothesis should be confirmed in further longitudinal studies.

Before the implementation of comprehensive stroke guidelines, Korea had the third highest rate of age-adjusted stroke mortality as shown by a 1997 OECD report (unpublished data). Even in the previous decade’s report, only 3% of acute ischemic stroke patients admitted to a hospital received IV thrombolysis [29], and the in-hospital mortality rate was found to be as high as 5.2% [30]. However, quality of stroke care in Korea has significantly advanced in the last decade, with the lowest current 30-day mortality rate among OECD countries [1]. Currently, 75% of neurology training hospitals have organized stroke centers and 85% of which have regular monitoring of performance measures to improve the quality of stroke management, such as screening of dysphagia, prophylaxis for deep vein thrombosis, and education for smoking cessation [31]. Moreover, a number of evidence-based quality indicators for stroke are extracted systematically and reported every month in each hospital [28]. As a result, the age-adjusted 1-year mortality rate of ischemic stroke patients has substantially decreased from 2002 to 2010 in a nationwide hospital-based registry report [32]. Moreover, the rate for IV thrombolysis treatment increased from 4.4% in 2002 to 6.0% in 2010.

The prognosis of off-hour presentation in patients with acute ischemic stroke has been scarcely reported [11]. In previous reports, off-hour admission in patients with ischemic stroke was not associated with worse prognosis at discharge [9], [11], [15], [17], [18], [20], which is in accordance with our present results. In contrast, Reeves et al showed that off-hour admission is associated with worse prognosis in patients with hemorrhagic or ischemic stroke. However, prognosis was only slightly worse in such patients, and was mainly attributed to hemorrhagic stroke presentation. In addition, in that study, the odds ratio of prognosis in the off-hour group declined with increasing duration of participation in the Get With The Guideline program. Taken together, these findings support the notion that organized stroke care may reduce the negative impact of off-hour treatment in patients with ischemic stroke.

**Figure 2:**
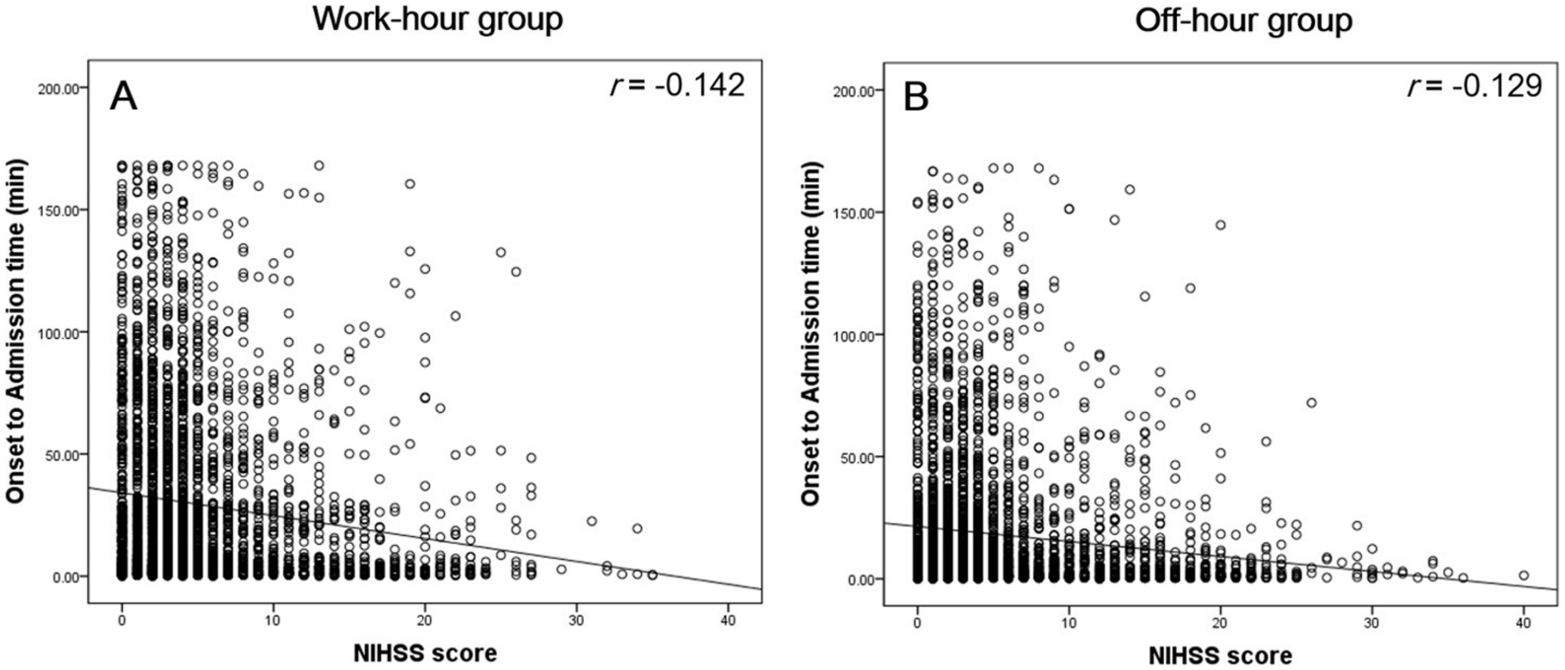
Correlation Between NIHSS Score and Onset-to-admission Time in Work-hour (A) and Off-hour Group (B).

Fang et al reported that stroke fatality was higher in patients admitted on weekend compared to weekday and the patients with more severe stroke tended to present to the hospital quickly, while patients with minor stroke or transient ischemic attack were more likely to delay admission until after the weekend [18]. As shown in Figure 2, there is a more pronounced inverse correlation between initial NHISS score and onset to admission time in work-hour admission group than in off-hour admission group. However, this tendency was not statistically significant when the correlation coefficients were compared (z statistics: −1.40, *P* = 0.16), which contradict the results by Fang et al. Moreover, previous reports suggested that stroke severity was higher in off-hour admission groups than in work-hour groups [9], [15], [20], which is congruous with our results. Patients with high NIHSS score were generally admitted without a prehospital delay during an off-hour period, which was reflected by the high rate of IV thrombolysis in the off-hour admission group. This may partly explain why patients in the off-hour group did not have a worse outcome compared to those in the work-hour group. To summarize, our data support these findings that stroke prognosis in patients admitted to the hospital during off-hours does not differ from that of patients admitted during work-hours irrespective of stroke severity and prehospital delay time.

In our data, prehospital delay times during work-hours were significantly longer than those during off-hours. Prehospital delay have been found to be associated with age, stroke severity, educational status, using ambulance, awareness of stroke symptoms or other behavioral factors [33], [34]. Although we do not considered factors influencing prehospital delay primarily, we can partially explain the observed prehospital delay as follows. Recent nationwide survey had been identified that public knowledge or awareness of stroke symptoms was suboptimal in Korea [35]. This can cause prehospital delay especially in stroke patients without neighboring family member in work-hours. When we analyzed patients admitted within 24 hours, prehospital delay times during work-hours were not significantly different compared with those during off-hours (4.5 hours vs. 3.8 hours, data was not shown). However, factors influencing prehospital delay in patients admitted during work-hours should be studied in detail using future trials.

Martinez-Martinez et al studied the association between in-hospital management and short-term outcome of stroke patients according to time of admission [11]. The author observed that the time to neuroimaging and door to needle time were not different between the two groups, which is partly reaffirmed by our data. These data support the notion that organized stroke care can be effectively implemented during off-hour periods.

Our study has several limitations. All of the 12 participating hospitals are tertiary teaching hospitals, located mainly in urban areas. Therefore, our results cannot be generalized to all populations in Korea. Moreover, patient exclusion owing to incomplete data capture may have led to selection bias. Although functional outcome and mortality data are highly comparable to well-established registry outcome data [36]. Selection bias for the missing mRS is the main limitation of our hospital –based registry which have to be resolved in future cohort study.

The following are some of the strengths of our study. Firstly, important outcome variables, such as stroke subtype, severity and risk factors, were adjusted in multiple comparisons between the two groups. Secondly, the 3-month mRS, which is the most widely used outcome variable in clinical trials, rather than discharge mRS, were used in our analysis. Importantly, a representative classification of off-hours and work-hours including statutory holidays better reflects the real world ‘off-hour’ effect due to poor hospital staffing.

## Conclusions

Off-hour admission was not associated with the 3-month unfavorable functional outcomes in acute ischemic stroke patients admitted at tertiary teaching hospitals in Korea. Our result provide evidence that off-hour effect can be overcome with well-organized stroke management strategies.

## Supporting Information

Table S1 **Baseline Characteristics of Patients with missing data.**
(DOCX)

Table S2 **Results of Univariable and Multivariable Analysis for Mortality at 3 Months.**
(DOCX)

Table S3 **Results of Univariable and Multivariable Shift Analysis of mRS Distribution to Worse Score at 3 Months.**
(DOCX)

Table S4 **Unfavorable Functional Outcome in Patients with IV Thrombolysis (n = 606).**
(DOCX)

Table S5 **Univariable and Multivariable Analysis for Unfavorable Functional Outcome at 3 Months in Patients Admitted Within 24 Hour after the Onset of Symptom.**
(DOCX)

## References

[1] Hong KS, Bang OY, Kang DW, Yu KH, Bae HJ, et al (2013) Stroke statistics in Korea: Part i. Epidemiology and risk factors: A report from the Korean Stroke Society and Clinical Research Center for Stroke (special report). Journal of Stroke 15: 2–20. 24324935 10.5853/jos.2013.15.1.2 PMC3779679

[2] Weimar C, Konig IR, Kraywinkel K, Ziegler A, Diener HC, German Stroke Study Collaboration (2004) Age and National Institutes of Health Stroke Scale score within 6 hours after onset are accurate predictors of outcome after cerebral ischemia: Development and external validation of prognostic models. Stroke 35: 158–162. 14684776 10.1161/01.STR.0000106761.94985.8B

[3] Adams HP Jr, Davis PH, Leira EC, Chang KC, Bendixen BH, et al (1999) Baseline NIH Stroke Scale score strongly predicts outcome after stroke: A report of the Trial of Org 10172 in Acute Stroke Treatment (TOAST). Neurology 53: 126–131. 10408548 10.1212/wnl.53.1.126

[4] Nedeltchev K, der Maur TA, Georgiadis D, Arnold M, Caso V, et al (2005) Ischaemic stroke in young adults: Predictors of outcome and recurrence. J Neurol Neurosurg Psychiatry 76: 191–195. 15654030 10.1136/jnnp.2004.040543 PMC1739502

[5] Capes SE, Hunt D, Malmberg K, Pathak P, Gerstein HC (2001) Stress hyperglycemia and prognosis of stroke in nondiabetic and diabetic patients: A systematic overview. Stroke 32: 2426–2432. 11588337 10.1161/hs1001.096194

[6] Kimberly WT, Lima FO, O’Connor S, Furie KL (2013) Sex differences and hemoglobin levels in relation to stroke outcomes. Neurology 80: 719–724. 23365064 10.1212/WNL.0b013e31828250ff PMC3589294

[7] Yahalom G, Schwartz R, Schwammenthal Y, Merzeliak O, Toashi M, et al (2009) Chronic kidney disease and clinical outcome in patients with acute stroke. Stroke 40: 1296–1303. 19182072 10.1161/STROKEAHA.108.520882

[8] Bejot Y, Aboa-Eboule C, Jacquin A, Troisgros O, Hervieu M, et al (2013) Stroke care organization overcomes the deleterious ‘weekend effect’ on 1-month stroke mortality: A population-based study. Eur J Neurol 20: 1177–1183. 23551852 10.1111/ene.12154

[9] Streifler JY, Benderly M, Molshatzki N, Bornstein N, Tanne D (2012) Off-hours admission for acute stroke is not associated with worse outcome–a nationwide Israeli stroke project. Eur J Neurol 19: 643–647. 22136626 10.1111/j.1468-1331.2011.03603.x

[10] Palmer WL, Bottle A, Davie C, Vincent CA, Aylin P (2012) Dying for the weekend: A retrospective cohort study on the association between day of hospital presentation and the quality and safety of stroke care. Arch Neurol 69: 1296–1302. 22777008 10.1001/archneurol.2012.1030

[11] Martinez-Martinez MM, Fernandez-Travieso J, Fuentes B, Ruiz-Ares G, Martinez-Sanchez P, et al (2012) Off-hour effects on stroke care and outcome in stroke centres. Eur J Neurol 19: 1140–1145. 22435893 10.1111/j.1468-1331.2012.03692.x

[12] Albright KC, Savitz SI, Raman R, Martin-Schild S, Broderick J, et al (2012) Comprehensive stroke centers and the ‘weekend effect’: The SPOTRIAS experience. Cerebrovasc Dis 34: 424–429. 23207423 10.1159/000345077 PMC3568158

[13] Ogbu UC, Westert GP, Slobbe LC, Stronks K, Arah OA (2011) A multifaceted look at time of admission and its impact on case-fatality among a cohort of ischaemic stroke patients. J Neurol Neurosurg Psychiatry 82: 8–13. 20667853 10.1136/jnnp.2009.202176

[14] McKinney JS, Deng Y, Kasner SE, Kostis JB (2011) Comprehensive stroke centers overcome the weekend versus weekday gap in stroke treatment and mortality. Stroke 42: 2403–2409. 21868723 10.1161/STROKEAHA.110.612317

[15] Haeusler KG, Gerischer LM, Vatankhah B, Audebert HJ, Nolte CH (2011) Impact of hospital admission during nonworking hours on patient outcomes after thrombolysis for stroke. Stroke 42: 2521–2525. 21799159 10.1161/STROKEAHA.110.612697

[16] Kazley AS, Hillman DG, Johnston KC, Simpson KN (2010) Hospital care for patients experiencing weekend vs weekday stroke: A comparison of quality and aggressiveness of care. Arch Neurol 67: 39–44. 20065127 10.1001/archneurol.2009.286

[17] Hoh BL, Chi YY, Waters MF, Mocco J, Barker FG 2nd (2010) Effect of weekend compared with weekday stroke admission on thrombolytic use, in-hospital mortality, discharge disposition, hospital charges, and length of stay in the nationwide inpatient sample database, 2002 to 2007. Stroke 41: 2323–2328. 20724715 10.1161/STROKEAHA.110.591081

[18] Fang J, Saposnik G, Silver FL, Kapral MK (2010) Association between weekend hospital presentation and stroke fatality. Neurology 75: 1589–1596. 21041782 10.1212/WNL.0b013e3181fb84bc

[19] Reeves MJ, Smith E, Fonarow G, Hernandez A, Pan W, et al (2009) Off-hour admission and in-hospital stroke case fatality in the get with the guidelines-stroke program. Stroke 40: 569–576. 18988914 10.1161/STROKEAHA.108.519355

[20] Jauss M, Oertel W, Allendoerfer J, Misselwitz B, Hamer H (2009) Bias in request for medical care and impact on outcome during office and non-office hours in stroke patients. Eur J Neurol 16: 1165–1167. 19469835 10.1111/j.1468-1331.2009.02656.x

[21] Saposnik G, Baibergenova A, Bayer N, Hachinski V (2007) Weekends: A dangerous time for having a stroke? Stroke 38: 1211–1215. 17347472 10.1161/01.STR.0000259622.78616.ea

[22] Hasegawa Y, Yoneda Y, Okuda S, Hamada R, Toyota A, et al (2005) The effect of weekends and holidays on stroke outcome in acute stroke units. Cerebrovasc Dis 20: 325–331. 16131801 10.1159/000087932

[23] Bell CM, Redelmeier DA (2004) Waiting for urgent procedures on the weekend among emergently hospitalized patients. Am J Med 117: 175–181. 15276596 10.1016/j.amjmed.2004.02.047

[24] Tung YC, Chang GM, Chen YH (2009) Associations of physician volume and weekend admissions with ischemic stroke outcome in Taiwan: A nationwide population-based study. Med care 47: 1018–1025. 19648828 10.1097/MLR.0b013e3181a81144

[25] Stroke Unit Trialists’ Collaboration (2000) Organised inpatient (stroke unit) care for stroke. Cochrane Database Syst Rev 2: CD000197. 10.1002/14651858.CD000197 10796318

[26] O’Rourke K, Walsh C (2010) Impact of stroke units on mortality: A Bayesian analysis. Eur J Neurol 17: 247–251. 19780805 10.1111/j.1468-1331.2009.02782.x

[27] Saposnik G, Kapral MK, Coutts SB, Fang J, Demchuk AM, et al (2009) Do all age groups benefit from organized inpatient stroke care? Stroke 40: 3321–3327. 19644068 10.1161/STROKEAHA.109.554907

[28] Kim BJ, Han MK, Park TH, Park SS, Lee KB, et al (2014) Current status of acute stroke management in Korea: A report on a multicenter, comprehensive acute stroke registry. Int J Stroke 9: 514–518. 24256115 10.1111/ijs.12199

[29] Yu KH, Bae HJ, Kwon SU, Kang DW, Hong KS, et al (2006) Analysis of 10,811 cases with acute ischemic stroke from Korean Stroke Registry: Hospital-based multicenter prospective registration study. J Kor Neurol Assoc 24: 535–543.

[30] Lee BC, Roh JK (2006) International experience in stroke registries: Korean Stroke Registry. Am J Prev Med 31: S243–245. 17178312 10.1016/j.amepre.2006.08.019

[31] Choi HY, Cha MJ, Nam HS, Kim YD, Hong KS, et al (2012) Stroke units and stroke care services in Korea. Int J Stroke 7: 336–340. 22510228 10.1111/j.1747-4949.2012.00788.x

[32] Jung KH, Lee SH, Kim BJ, Yu KH, Hong KS, et al (2012) Secular trends in ischemic stroke characteristics in a rapidly developed country: Results from the Korean Stroke Registry study (secular trends in Korean stroke). Circ Cardiovasc Qual Outcomes 5: 327–334. 22474244 10.1161/CIRCOUTCOMES.111.963736

[33] Harraf F, Sharma AK, Brown MM, Lees KR, Vass RI, et al (2002) A multicentre observational study of presentation and early assessment of acute stroke. BMJ 6: 17. 10.1136/bmj.325.7354.17 PMC116666 12098723

[34] Kwan J, Hand P, Sandercock P (2004) A systematic review of barriers to delivery of thrombolysis for acute stroke. Age Ageing 33: 116–121. 14960425 10.1093/ageing/afh064

[35] Kim YS, Park SS, Bae HJ, Cho AH, Cho YJ, et al (2011) Stroke awareness decreases prehospital delay after acute ischemic stroke in Korea. BMC Neurology 11: 2. 21211051 10.1186/1471-2377-11-2 PMC3023649

[36] Fonarow GC, Pan W, Saver JL, Smith EE, Reeves MJ, et al (2012) Comparison of 30-day mortality models for profiling hospital performance in acute ischemic stroke with vs without adjustment for stroke severity. JAMA 308: 257–264. 22797643 10.1001/jama.2012.7870

