# Supplementary material for: Off-Hour Effect on 3-Month Functional Outcome after Acute Ischemic Stroke: A Prospective Multicenter Registry
Source: PLoS One. 2014 Aug 28;9(8):e105799. doi: 10.1371/journal.pone.0105799 (PMC4148337; doi:10.1371/journal.pone.0105799)
Supplement: Table S1 — Baseline Characteristics of Patients with missing data. (DOCX) [file pone.0105799.s001.docx]

**Table S1. Baseline Characteristics of Patients with missing data**

|  | Patients with mRS  (n=7075) | Patients without mRS  (n=532) | *P* value |
| --- | --- | --- | --- |
| Age, year | 67.5 ± 13.0 | 68.7 ± 13.5 | 0.051 |
| Male | 4146 (58.6%) | 300 (56.4%) | 0.312 |
| Risk factor (%) |  |  |  |
| Previous stroke | 1519 (21.5%) | 125 (23.5%) | 0.273 |
| Hypertension | 4927 (69.6%) | 360 (67.7%) | 0.341 |
| Diabetes | 2379 (33.6%) | 193 (36.3%) | 0.212 |
| Hyperlipidemia | 2334 (33.0%) | 238 (44.7%) | 0.001 |
| Current Smoking | 1874 (26.5%) | 137 (25.8%) | 0.711 |
| TIA presentation and Stroke subtype, n (%) |  |  | 0.098 |
| TIA presentation | 35 (0.5%) | 0 (0%) |  |
| LAA | 2629 (36.9%) | 197 (37.0%) |  |
| SVO | 1365 (19.3%) | 75 (14.1%) |  |
| CE | 1415 (20.0%) | 122 (22.9%) |  |
| SOE | 176 (2.5%) | 8 (1.5%) |  |
| SUE | 1475 (20.8%) | 130 (24.4%) |  |
| NIHSS, median (IQR) | 3 (5) | 6 (10) | <0.001 |
| Prehospital delay (hour) | 1.1 (1.1) | 1.2 (1.4) | 0.302 |
| Onset to needle time | 115 (63) | 124 (75) | 0.125 |
| Door to needle time | 45 (22) | 39 (29) | 0.066 |
| IV rtPA | 606 (8.6%) | 57 (10.7%) | 0.090 |
| Off-hour (vs. Work-hour) | 3473 (49.1%) | 279 (52.4) | 0.135 |

Abbreviations are presented in the previous table.
