# Supplementary material for: Off-Hour Effect on 3-Month Functional Outcome after Acute Ischemic Stroke: A Prospective Multicenter Registry
Source: PLoS One. 2014 Aug 28;9(8):e105799. doi: 10.1371/journal.pone.0105799 (PMC4148337; doi:10.1371/journal.pone.0105799)
Supplement: Table S2 — Results of Univariable and Multivariable Analysis for Mortality at 3 Months. (DOCX) [file pone.0105799.s002.docx]

**Table S2**. **Results of Univariable and Multivariable Analysis for Mortality at 3 Months.**

|  | Univariable OR (95% CI) | *P value* | Multivariable (95% CI) |
| --- | --- | --- | --- |
| Age, year | 1.07 (1.06-1.08) | <0.001 | 1.05 (1.03-1.06) |
| Male | 0.66 (0.54-0.80) | <0.001 | 1.05 (0.73-1.53) |
| Risk factor (%) |  |  |  |
| Previous stroke | 1.66 (1.34-2.06) | <0.001 | 1.39(0.95-2.04) |
| Hypertension | 1.26 (1.01-1.57) | 0.043 | 1.32 (0.88-1.99) |
| Diabetes | 1.11 (0.90-1.36) | 0.320 |  |
| Hyperlipidemia | 0.68 (0.55-0.85) | 0.001 | 0.59 (0.38-0.91) |
| Current Smoking | 0.64 (0.50-0.82) | <0.001 | 1. 03 (0.64-1.67) |
| Stroke subtype, n (%) |  |  |  |
| LAA | 5.00 (2.74-9.11) | <0.001 | 4.92 (1.50-16.19) |
| SVO | 1.0 (reference) | - | 1.0 (reference) |
| CE | 14.94 (8.27-26.99) | <0.001 | 6.60 (1.99-21.83) |
| SOE | 14.83 (7.11-30.91) | <0.001 | 25.30 (6.28-101.91) |
| SUE | 10.71 (5.90-19.46) | <0.001 | 8.02 (2.42-26.53) |
| NIHSS at admission, score | 1.18 (1.16-1.19) | <0.001 | 0.14 (1.11-1.16) |
| Prehospital delay (hour) | 0.99 (1.00-1.00) | <0.001 | 1.00 (0.99-1.00) |
| IV rtPA | 1.71 (1.24-2.37) | 0.001 | 0.81 (0.54-1.20) |
| Off-hour (vs. Work-hour) | 1.24 (1.02-1.51) | 0.033 | 1.09 (0.77-1.54) |

Abbreviations are presented in the previous table.
