# Supplementary material for: Off-Hour Effect on 3-Month Functional Outcome after Acute Ischemic Stroke: A Prospective Multicenter Registry
Source: PLoS One. 2014 Aug 28;9(8):e105799. doi: 10.1371/journal.pone.0105799 (PMC4148337; doi:10.1371/journal.pone.0105799)
Supplement: Table S3 — Results of Univariable and Multivariable Shift Analysis of mRS Distribution to Worse Score at 3 Months. (DOCX) [file pone.0105799.s003.docx]

**Table S3**. **Results of Univariable and Multivariable Shift Analysis of mRS Distribution to Worse Score at 3 Months.**

|  | Univariable OR (95% CI) | *P value* | Multivariable OR (95% CI) |
| --- | --- | --- | --- |
| Age, year | 1.05 (1.05-1.05) | <0.001 | 1.04 (1.03-1.05) |
| Male | 0.60 (0.55-0.65) | <0.001 | 0.79 (0.67-0.92) |
| Risk factor (%) |  |  |  |
| Previous stroke | 2.30 (2.08-2.55) | <0.001 | 1.69 (1.41-2.02) |
| Hypertension | 1.43 (1.31-1.56) | <0.001 | 1.02 (0.87-1.21) |
| Diabetes | 1.36 (1.25-1.49) | <0.001 | 1.33 (1.13-1.56) |
| Hyperlipidemia | 0.97 (0.89-1.06) | 0.522 |  |
| Current Smoking | 0.65 (0.59-0.71) | <0.001 | 1.15 (0.95-1.39) |
| TIA presentation and Stroke subtype, n (%) |  |  |  |
| TIA presentation | 0.10 (0.04-0.26) | <0.001 | 0.00 (0.00-0.00) |
| LAA | 1.81 (1.61-2.03) | <0.001 | 1.28 (1.03-1.58) |
| SVO | 1.0 (reference) | - | 1.0 (reference) |
| CE | 2.69 (2.35-3.07) | <0.001 | 1.00 (0.78-1.27) |
| SOE | 1.30 (0.98-1.72) | 0.063 | 1.91 (1.19-3.06) |
| SUE | 1.68 (1.47-1.91) | 0.067 | 0.96 (0.75-1.22) |
| NIHSS at admission, score | 1.29 (1.27-1.30) | <0.001 | 1.26 (1.24-1.28) |
| Prehospital delay (hour) | 1.00 (1.00-1.00) | 0.075 | 1.00 (1.00-1.00) |
| IV rtPA | 1.34 (1.14-1.57) | <0.001 | 0.55 (0.46-0.67) |
| Off-hour (vs. Work-hour) | 0.7 (0.89-1.05) | 0.461 | 0.90 (0.78-1.05) |

Abbreviations are presented in the previous table.
