# Supplementary material for: Off-Hour Effect on 3-Month Functional Outcome after Acute Ischemic Stroke: A Prospective Multicenter Registry
Source: PLoS One. 2014 Aug 28;9(8):e105799. doi: 10.1371/journal.pone.0105799 (PMC4148337; doi:10.1371/journal.pone.0105799)
Supplement: Table S4 — Unfavorable Functional Outcome in Patients with IV Thrombolysis (n = 606). (DOCX) [file pone.0105799.s004.docx]

**Table S4**. **Unfavorable Functional Outcome in Patients with IV Thrombolysis (n=606).**

|  | Univariable OR (95% CI) | *P value* | Multivariable OR (95% CI) |
| --- | --- | --- | --- |
| Age, year | 1.06 (1.05-1.08) | <0.001 | 1.05 (1.03-1.07) |
| Male | 0.58 (0.42-0.81) | 0.001 | 0.94 (0.62-1.41) |
| Risk factor (%) |  |  |  |
| Previous stroke | 1.29 (0.86-1.95) | 0.216 |  |
| Hypertension | 1.31 (0.93-1.84) | 0.128 |  |
| Diabetes | 1.41 (0.98-2.05) | 0.068 | 1.30 (0.84-2.01) |
| Hyperlipidemia | 0.98 (0.69-1.37) | 0.887 |  |
| Current Smoking | 0.50 (0.35-0.74) | <0.001 | 0.71 (0.44-1.15) |
| Stroke subtype, n (%) |  |  |  |
| LAA | 2.12 (1.13-4.00) | <0.019 | 1.31 (0.66-2.60) |
| SVO | 1.0 (reference) | - | 1.0 (reference) |
| CE | 3.56 (1.92-6.60) | <0.001 | 0.98 (0.49-2.00) |
| SOE | 1.23 (0.33-4.52) | 0.756 | 1.61 (0.38-6.88) |
| SUE | 2.41 (1.27-4.59) | 0.007 | 1.06 (0.52-2.18) |
| NIHSS at admission, score | 1.20 (1.16-1.24) | <0.001 | 1.18 (1.14-1.23) |
| Prehospital delay, hour | 0.91 (0.57-1.43) | 0.667 |  |
| Onset to Needle time, min | 1.00 (1.00-1.01) | 0.611 |  |
| Door to Needle time, min | 1.00 (1.00-1.00) | 0.618 |  |
| Off-hour (vs. Work-hour) | 0.79 (0.57-1.09) | 0.154 | 0.85 (0.58-1.25) |

Abbreviations are presented in the previous table.
