# Supplementary material for: Off-Hour Effect on 3-Month Functional Outcome after Acute Ischemic Stroke: A Prospective Multicenter Registry
Source: PLoS One. 2014 Aug 28;9(8):e105799. doi: 10.1371/journal.pone.0105799 (PMC4148337; doi:10.1371/journal.pone.0105799)
Supplement: Table S5 — Univariable and Multivariable Analysis for Unfavorable Functional Outcome at 3 Months in Patients Admitted Within 24 Hour after the Onset of Symptom. (DOCX) [file pone.0105799.s005.docx]

**Table S5**. **Univariable and Multivariable Analysis for Unfavorable Functional Outcome at 3 Months in Patients Admitted Within 24 Hour after the Onset of Symptom.**

|  | Unfavorable outcome  (n=1889) | Favorable outcome  (n=3051) | Univariable analysis | | Multivariable analysis | |
| --- | --- | --- | --- | --- | --- | --- |
|  |  |  | OR (95% CI) | *P* | *aOR (95% CI) | *P* |
| Age, year | 72.8 (±11.7) | 63.3 (±12.9) | 1.06 (1.05-1.06) | <0.001 | 1.04 (1.04-1.07) | <0.001 |
| Male | 934 (49.4) | 1987 (65.1) | 0.52 (0.47-0.59) | <0.001 | 0.68 (0.52-0.88) | 0.003 |
| Risk factor (%) |  |  |  |  |  |  |
| Previous stroke | 568 (30.1) | 506 (16.6) | 2.16 (1.89-2.48) | <0.001 | 1.93(1.46-2.55) | <0.001 |
| Hypertension | 1386 (73.4) | 2045 (67.0) | 1.36 (1.19-1.54) | <0.001 | 0.87 (0.66-1.15) | 0.337 |
| Diabetes | 677 (35.8) | 876 (28.7) | 1.39 (1.23-1.57) | <0.001 | 1.19 (0.92-1.55) | 0.189 |
| Hyperlipidemia | 606 (32.1) | 1029 (33.7) | 0.93 (0.82-1.05) | 0.232 |  |  |
| Current Smoking | 371 (19.6) | 911 (29.9) | 0.57 (0.50-0.71) | <0.001 | 1.00 (0.73-1.38) | 0.989 |
| TIA presentation and Stroke subtype, n (%) |  |  |  |  |  |  |
| TIA presentation | 3 (0.2) | 27 (0.9) | 0.42 (0.13-1.41) | 0.161 | 0.01 (1.00-1.00) | 1.000 |
| LAA | 660 (34.9) | 982 (32.2) | 2.56 (2.12-3.09) | <0.001 | 1.50 (1.06-2.13) | 0.022 |
| SVO | 189 (10.0) | 720 (23.6) | 1.0 (referent) | - | 1.0 (referent) | - |
| CE | 577 (30.5) | 584 (19.1) | 3.76 (3.09-4.58) | <0.001 | 0.97 (0.66-1.43) | 0.877 |
| SOE | 44 (2.3) | 82 (2.7) | 2.04 (1.37-3.05) | <0.001 | 4.54 (2.22-9.27) | <0.001 |
| SUE | 416 (22.0) | 656 (21.5) | 2.42 (1.97-2.96) | <0.001 | 0.98 (0.67-1.45) | 0.923 |
| NIHSS score at admission |  |  |  |  |  |  |
| Mean (±SD) | 10.1 (±7.1) | 3.3 (±3.6) | 1.28 (1.26-1.31) | <0001 | 1.25 (1.21-1.28) | <0.001 |
| Median (IQR) | 9 (11) | 2 (3) |  | <0.001 |  |  |
| Prehospital delay (hour) | 6.4 (±6.4) | 6.6 (±6.3) | 1.00 (0.99-1.01) | 0.461^†^ | 1.00 (0.99-1.02) | 0.639 |
| Onset to Needle time (min)  Median (IQR) | 115 (66) | 115 (63) |  | 0.994^†^ |  |  |
| Door to Needle time (min)  Median (IQR) | 45 (24) | 45 (22) |  | 0.680^†^ |  |  |
| IV rtPA | 276 (38.3) | 321 (32.4) | 1.30 (1.06-1.59) | 0.011 | 0.54 (0.40-0.73) | <0.001 |
| Off-hour (vs. Work-hour) | 1030 (54.5) | 1683 (55.2) | 0.98 (0.87-1.09) | 0.662 | 0.89 (0.70-1.13) | 0.335 |

Abbreviations are presented in the previous table.

* aOR: adjusted odds ratio

^†^*Mann-Whitney* U test
